# Options for reducing HIV transmission related to the dead space in needles and syringes

**DOI:** 10.1186/s12954-017-0207-5

**Published:** 2018-01-15

**Authors:** William A. Zule, Poonam G. Pande, David Otiashvili, Georgiy V. Bobashev, Samuel R. Friedman, V. Anna Gyarmathy, Don C. Des Jarlais

**Affiliations:** 1 RTI International, 3040 E. Cornwallis Road, PO Box 12194, Research Triangle Park, NC 2709-2194 USA; 2 Integrated CMC Solutions, LLC, 1289 Fordham Blvd Suite 201, Chapel Hill, NC 27514 USA; 3 Addiction Research Center—Alternative Georgia, 14A Nutsubidze Street, Office 2, 0177 Tbilisi, Georgia; 4 Institute of Infectious Disease Research, National Development and Research Institutes, 71 West 23d Street, 4th floor, New York, NY 10010 USA; 5 Johns Hopkins Bloomberg School of Public Health, Johns Hopkins University, Baltimore, MD USA; 6 Mount Sinai Beth Israel, 39 Broadway, Suite 530, New York, NY 10006 USA

**Keywords:** HIV, Hepatitis C virus, Dead space, Syringes, Needles, People who inject drugs, Needle and syringe programs

## Abstract

**Background:**

When shared by people who inject drugs, needles and syringes with different dead space may affect the probability of HIV and hepatitis C virus (HCV) transmission differently.

**Methods:**

We measured dead space in 56 needle and syringe combinations obtained from needle and syringe programs across 17 countries in Europe and Asia. We also calculated the amounts of blood and HIV that would remain in different combinations following injection and rinsing.

**Results:**

Syringe barrel capacities ranged from 0.5 to 20 mL. Needles ranged in length from 8 to 38 mm. The average dead space was 3 μL in low dead space syringes with permanently attached needles, 13 μL in high dead space syringes with low dead space needles, 45 μL in low dead space syringes with high dead space needles, and 99 μL in high dead space syringes with high dead space needles. Among low dead space designs, calculated volumes of blood and HIV viral burden were lowest for low dead space syringes with permanently attached needles and highest for low dead space syringes with high dead space needles.

**Conclusion:**

The dead space in different low dead space needle and syringe combinations varied substantially. To reduce HIV transmission related to syringe sharing, needle and syringe programs need to combine this knowledge with the needs of their clients.

**Electronic supplementary material:**

The online version of this article (10.1186/s12954-017-0207-5) contains supplementary material, which is available to authorized users.

## Background

HIV and hepatitis C virus (HCV) among people who inject drugs (PWID) are serious global health problems [1, 2]. In many countries, direct sharing of needles and syringes and syringe-mediated drug sharing account for most HIV and HCV infections among PWID. With adequate funding, culturally appropriate needle and syringe programs (NSPs) can reduce direct syringe sharing and HIV transmission [3, 4]. The World Health Organization (WHO) and UNAIDS have endorsed comprehensive HIV prevention packages for PWID that include NSP [5, 6]. In 2012, the WHO recommended that NSPs offer their clients low dead space (LDS) syringes [5, 7], which may reduce the survival of HIV and HCV [8, 9] as well as reduce transmission risk [10–12]. This recommendation was based on LDS syringes with permanently attached needles.

**Fig. 1:**
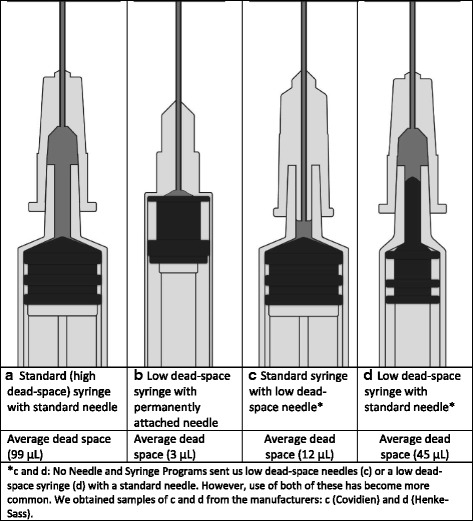
Needle and syringe designs that affect dead space. **a** Standard (high dead space) syringe with standard needle. Average dead space (99 μL). **b** Low dead space syringe with permanently attached needle. Average dead space (3 μL). **c** Standard syringe with low dead space needle. No needle and syringe programs sent us low dead space needles (**c**) or a low dead space syringe (**d**) with a standard needle. However, use of both of these has become more common. We obtained samples of **c** and **d** from the manufacturers: **c** Covidien and **d** Henke-Sass

However, LDS syringes with permanently attached needles are not acceptable to many PWID. In the years since the WHO issued the recommendation, several additional LDS needle and syringe options have become more widely available (see Fig. 1). To date, few published studies have examined how the attributes of these newer LDS options, which are likely to increase their acceptability to PWID, may affect their potential impact on HIV and HCV transmission.

When the plunger is fully depressed, all needle and syringe combinations retain some residual fluid in the needle itself, the hub of the needle, and the nozzle of the syringe. This void area has been labeled “dead space” [13]. When PWID share needles and syringes, the volume of dead space is an important determinant of the volume of blood that is transferred from one PWID to another [14]. The volume of inoculum (i.e., blood) multiplied by the HIV viral load (HIV viral particles per milliliter) equals the HIV viral burden (i.e., number of HIV particles) in an exposure [15, 16]. HIV treatment as prevention is based on the concept of reducing the HIV viral burden in exposures [17] by reducing the HIV viral load. LDS syringes reduce the HIV viral burden by reducing the volume of inoculum in an exposure [12, 14]. In both cases, the reduction in the HIV viral burden is hypothesized to reduce the probability of HIV transmission associated with an exposure. However, evidence regarding the impact of reduced dead space on HIV and HCV transmission remains circumstantial [8, 12, 18–21], while evidence from randomized controlled trials supports the efficacy of HIV treatment as prevention [20, 21].

In simulation studies, LDS syringes with permanently attached needles substantially reduced the spread of HIV among PWID [10, 12, 22]. Evidence suggests that LDS syringes with permanently attached needles may also reduce HCV transmission risk [9, 23–25]. Two recent studies examined the effects of other LDS needle and syringe designs on HIV and HCV survival [18, 19].

PWID around the world use a variety of needles and syringes that vary in size and design [26, 27]. In some countries, PWID inject home-produced drugs that require injecting volumes of fluid greater than 1 mL [28, 29]; consequently, they use larger (2 through 10 mL) syringes. Whereas in other places, many PWID inject heroin and prefer 1 mL syringes [26]. Also, many PWID who inject in shallow veins prefer short thin needles [30]; PWID who inject in deep veins require long thick needles [31, 32]. WHO recommends that NSPs offer a range of needles and syringes that meet the needs of local PWID [5].

Our previous papers on this topic assumed that syringe barrel capacity has little effect on dead space volume [14, 33]. This assumption was based on the fact that the tips of the nozzles of all high dead space (HDS) syringes have the same external diameter and the (Luer) taper of the external walls of syringe nozzles is standard. We previously overlooked the possibility that syringe nozzles may vary in height, wall thickness, and taper of the interior wall, which would affect the bore (i.e., internal diameter) of a nozzle. Variations in any of these dimensions could affect dead space, and they may have profound implications for new designs to reduce dead space in needles and syringes. This paper empirically tests those assumptions, and it explores the implications of violations of the assumptions for new designs to reduce dead space. In this article, we examine the characteristics of needles and syringes distributed by 22 NSPs across Europe and Asia. We also measured the dead space in different HDS and LDS syringe combinations and used statistical models to assess the effects of needle length, needle gauge, syringe barrel capacity, and syringe design on dead space volume. Finally, we adapted formulas for calculating serial dilutions to calculate the effects of dead space volume in the different needle and syringe combinations on the volumes of blood and HIV viral burden following the injection process and rinsing with water.

## Methods

### Survey and syringe collection

In 2009 and 2010, we contacted representatives of the Eurasian Harm Reduction Network and the Asian Harm Reduction Network, posted notices regarding the project on needle discussion list serves, and approached participants at various conferences, such as the International Harm Reduction Conference, the International AIDS Conference, and the National Conference on Injecting Drug Use. Our rationale for focusing on the region of Central/Eastern Europe and Asia was the high prevalence of injection drug use and high prevalence of HIV among PWID in these regions. We also worked through our contacts in the field. In addition, we contacted authors of papers on HIV among PWID in cities and countries where we did not have any contacts. In some instances, these authors provided information. In instances in which authors were not familiar with the types of syringes used by PWID in their city or country, they were often able to put us in contact with people who had this knowledge. Once we identified a potentially knowledgeable source, we emailed them a cover letter that explained the study and a brief survey formatted as a fillable PDF, which could be completed and returned via email (Additional file 1). The cover letter and survey were available in English and Russian. We are unable to calculate response rates for the survey because there was no sampling frame from which to select cities.

In addition to completing the survey, we asked respondents to send us five new needles and syringes of each combination used by PWID in their city or area. In situations where it was not practical to send syringes, we asked respondents to send digital pictures of syringes. Respondents received US$50 as compensation for their time and effort. To reduce the burden of sending syringes, respondents who sent syringes were given a prepaid code for a delivery service (e.g., United Parcel Service, DHL, or Federal Express) that operated in their area. Information provided by respondents was done so in their professional capacity as NSP operators, researchers, or service providers.

At the time of the study, we also purchased samples of LDS detachable needles from one manufacturer (Covidien). We were unable to identify any other manufacturer of LDS detachable needles at the time.

### Survey responses

**Fig. 2:**
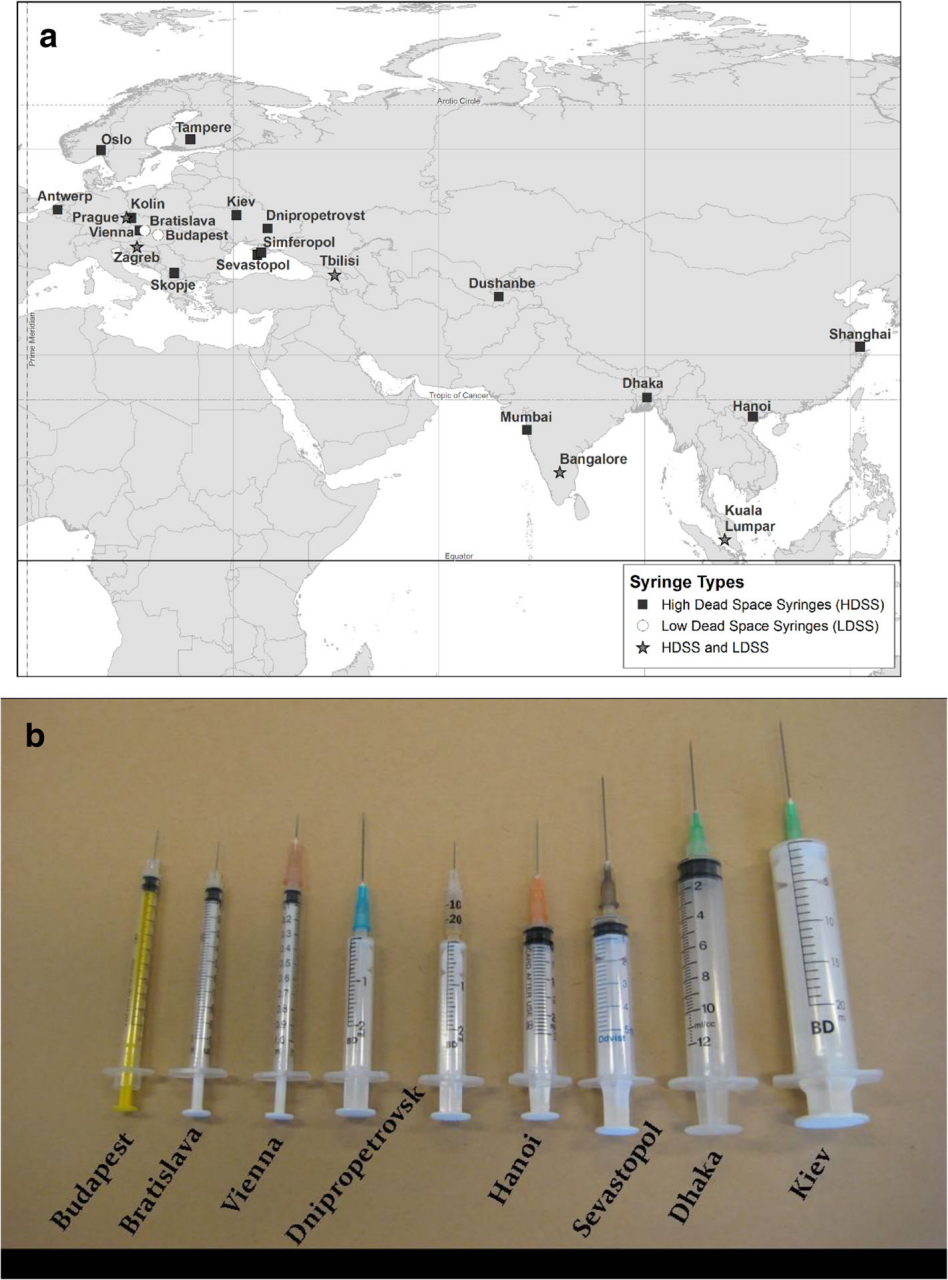
**a** City, country, and types of syringes sent. **b** Examples of needles and syringes from different cities

Altogether, we received completed surveys from NSPs in over 70 cities in 30 countries. This report is limited to NSPs in 22 cities in 17 countries (Fig. 2) that sent us needles and syringes. The needles and syringes were produced by 19 different manufacturers. Two thirds of the needles and syringes were produced by four manufacturers: Terumo (24.6%), BD (24.4%), Braun (9.0%), and Nipro (7.6%). Two cities sent only LDS syringes with permanently attached needles, five cities sent LDS syringes with permanently attached needles and HDS syringes with HDS needles, and 15 cities sent only HDS syringes with HDS needles (Fig. 2).

### Attributes of needles and syringes

Needle length, needle gauge, and LDS design are the attributes of needles that are most likely to affect dead space volume. In the USA, needle length is measured in inches and thickness is measured in gauge, with larger numbers representing thinner needles. In other parts of the world, needle length and needle gauge are measured in millimeters. Needle gauge refers to the outside diameter of the needle. The shaft of a needle may be thick-walled or thin-walled; consequently, needles with the same outside diameter may have a different bore (i.e., inside diameter). Barrel capacity and design (LDS or HDS) are the attributes of syringes that are most likely to influence dead space volume.

We received needles in seven lengths, ranging from 8 mm (5/16 in.) to 38 mm (1 1/2 in.), and 10 gauges ranging from 0.30 mm (30 G) to 0.90 mm (20 G). These represented 23 distinct combinations of needle length and gauge. We received seven different capacities of syringe barrels ranging from 0.5 to 20 mL.

### Measurement of dead space

We used the gravimetric method [34] to measure the dead space in needles and syringes. We weighed each needle and syringe combination new and dry. Then, we drew excess deionized water into the syringe, expelled any air bubbles in the syringe or needle hub, and adjusted the volume to the labeled capacity of the syringe. The syringe needle combination with water was then weighed. The water was expelled all the way and the syringe needle combination was weighed to determine the amount of water retained in the dead space. This process was repeated three times for standard needle and syringe combinations. To reduce the effect of outliers caused by small volumes of dead space, we repeated the process five times with each LDS syringe with a permanently attached needle.

### Procedures for assessing the effect of needle length, needle gauge, syringe barrel capacity, and design on dead space volume

Similar to the process described above, dead space was calculated for three syringes of each barrel size (1, 2, 3, 5, 10, and 20 mL) with a 25-gauge (5/8-in.) needle attached and for three needles of each length and gauge attached to a 5-mL syringe.

### Statistical analysis of factors contributing to dead space

We performed multiple linear regression using SPSS version 23 (IBM Corporation) to assess the independent effects of syringe design, barrel capacity, needle length, and needle gauge on dead space volume in all syringes and in standard needle and syringe combinations.

### Calculating the effect of dead space on volume of blood and HIV viral burden retained in syringes

We used mathematical formulas to calculate the volume of blood and HIV viral burden in LDS syringes with permanently attached needles, LDS syringes with an HDS needle attached, HDS syringes with an LDS needle attached, and HDS syringes with an HDS needle attached. The formulas represent variations of the common laboratory formula used for calculating serial dilutions [35]. In a previous experimental study of blood retained in syringes following simulated injection and rinsing, the calculated volume of blood retained fell within 95% confidence intervals for volumes observed in the experiments [14, 33]. For the HDS syringes with HDS needles, we limited the calculations to 1- through 5-mL syringes because 10- and 20-mL syringes are used primarily for preparing drug solutions and less often for injecting [26]. The formulas are shown along with the results in Fig. 4.

## Results

### Dead space measurements

**Fig. 3:**
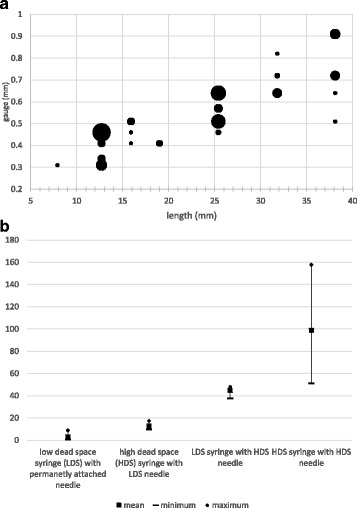
**a** Distribution of needle lengths and gauges. The size of the dots indicates the number of different needles of a specific length and gauge that we received. The smallest dots represent 1 needle and the largest dot represents 22 needles. **b** Dead space in needle and syringe combinations, by design

We measured the dead space in 56 different needle and syringe combinations (i.e., needle length, needle gauge, barrel capacity, and manufacturer) that we obtained from NSP (see examples in Fig. 3a) and in two other LDS syringe designs—one that a researcher sent us and one that we purchased from the manufacturer. The mean dead space ranged from 3.0 μL in LDS syringes with permanently attached needles to 98.9 μL in HDS syringes with HDS needles (Table 1). The mean dead space by barrel capacity in HDS needles with HDS syringes ranged from 81 μL (S.D. 14.0) in the 1-mL syringes to 132 μL (S.D. 15.9) in the 20-mL syringes.

**Table 1:** Dead space in high and low dead space needle and syringe combinations

Syringe	Needle	Mean	Standard deviation
Dead space	Type/dead space		
Low	Low	3.0	2.0
High	Low	13.0	1.8
Low	High	45.0	4.2
High	High	98.9	21.0

### Factors influencing dead space in needle and syringe combinations

#### Low dead space needles and syringes

We tested 10 different 1-mL and one 0.5-mL syringes with permanently attached needles, two different LDS needles with HDS syringes, and one LDS syringe with an HDS needle. Dead space was somewhat higher in the 1-mL syringes (3.1 μL, S.D. 2.0 μL) than in the 0.5-ml syringes (1.7 μL, S.D. 0.9 μL). The mean dead space in the Monoject™ LDS needles attached to special 1-, 2-, 3-, and 5-mL Monoject™ syringes was 12.1 μL (S.D. 1.8 μL). The mean dead space in the 1-mL LDS syringes with HDS needles was 44.6 μL (S.D. 4.1 μL).

#### HDS syringes with HDS needles

We tested 45 different standard high dead space needle and syringe combinations that were produced by 19 different manufacturers. In bivariate analyses, larger barrel capacity, longer needle length, and thicker needle gauge were all significantly associated with a greater volume of dead space. In multiple linear regression models, barrel capacity explained 26% of the variance, whereas needle length and gauge explained around 1% (Table 2).

**Table 2:** The effects of syringe design, barrel capacity, needle length, and needle gauge on dead space in needle and syringe combinations

	Linear regression			Multiple linear regression of all syringes			Multiple linear regression of high dead space syringes (HDS) with HDS needles only		
Variable	*β* ^a^	*R* ^2^	*p* value	*β*	*R*^2^ change	*p* value	*β*	*R*^2^ change	*p* value
Syringe design^b^	0.877	0.769	< 0.001	0.797	0.769	< 0.001			
Barrel capacity (mL)	0.512	0.262	< 0.001	0.272	0.060	< 0.001	0.537	0.262	< 0.001
Needle length (mm)	0.195	0.038	0.001	0.112	0.001	0.014	0.215	0.013	0.044
Needle gauge (mm)	0.211	0.044	< 0.001	− 0.109	0.002	0.036	− 0.196	< 0.001	0.022

^a^Coding for syringe design: low dead space syringe with permanently attached needle = 0; high dead space syringe with high dead space needle = 1

^*b*^*β* = standardized regression coefficient

#### Calculated impact of needle and syringe dead space and rinsing with water on volumes of blood and HIV viral burden following injection and rinsing

**Fig. 4:**
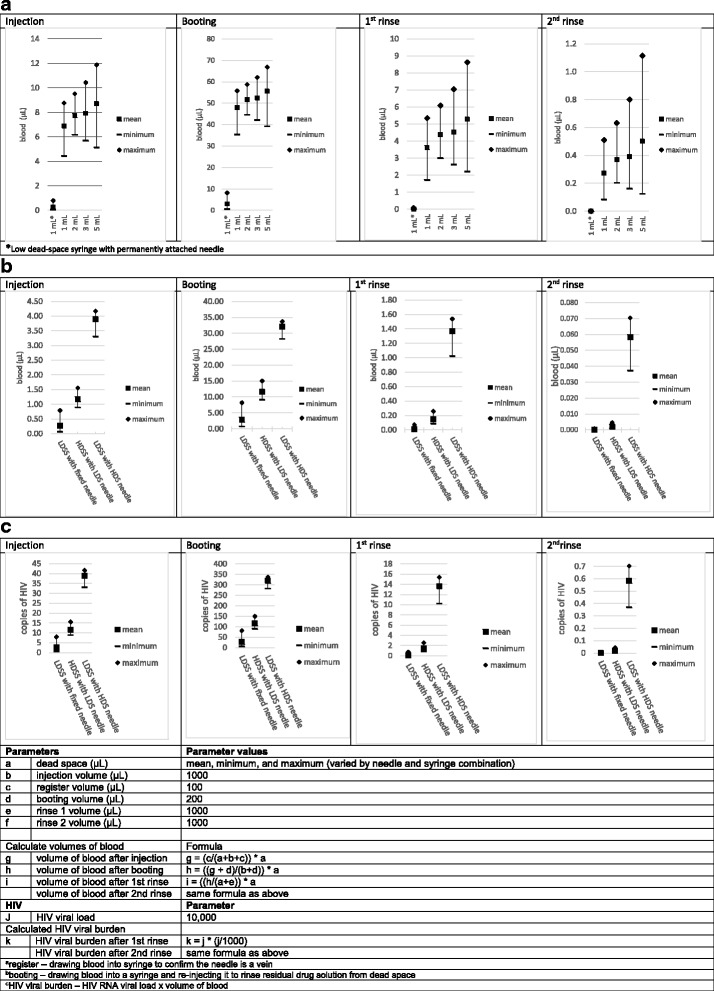
**a** Volume of blood by barrel capacity and low dead space permanently attached needle or high dead space detachable needle after injection, booting, first rinse, and second rinse. **b** Volume of blood by low dead space design after injection, booting, first rinse, and second rinse. **c** HIV viral burden after injection, booting, first rinse, and second rinse. The asterisk indicates low dead space syringe with permanently attached needle

The effects of dead space on the volume of blood and the HIV viral burden retained in a syringe after injection and rinsing are shown in Fig. 4a–c—indicating that for detachable combinations, needle and syringe combination matters more than barrel size. As Fig. 4a shows, the volume of blood after injection, booting (i.e., drawing blood into the syringe and reinjecting it to rinse residual drug solution from the dead space), first rinse, and second rinse was significantly lower (close to 0 μL) in LDS syringes with permanently attached needles than in any size of HDS syringes, and HDS syringe sizes were not significantly different from each other. On the other hand, Fig. 4b shows that LDS syringes with permanently attached needles retained significantly less blood than HDS syringes with LDS needles, and the latter had a significantly lower volume of blood than LDS syringes with HDS needles, respectively, after each step. Moreover, Fig. 4c shows that (with the parameters set for the model—see bottom of figure) the HIV viral burden was significantly lower in LDS syringes with permanently attached needles than in HDS syringes with LDS needles, and it was considerably higher in LDS syringes with HDS needles than in HDS syringes with LDS needles, after each step.

In these examples, we used an HIV viral load of 10,000 copies/mL. During chronic HCV infection, several studies suggest that the HCV viral load is approximately one million copies/mL [36–38]. Using an HCV viral load of one million would result in an HCV viral burden 100 times greater than the calculated HIV viral burden estimates in Fig. 4c.

## Discussion

Ideally, PWID would never share syringes, but this would likely require dramatic increases in funding needle and syringe programs, unrestricted over-the-counter syringe sales, and full decriminalization of needles and syringes. Even then, substantial minorities of PWID may continue to engage in syringe-mediated drug sharing. Stop-gap measures that reduce the risk of HIV and HCV transmission associated with these practices can help slow transmission while the world strives to implement the changes necessary to achieve these goals. Increasing the availability and use of LDS needles and syringes represents one such measure.

In computations, the calculated volumes of blood and HIV viral burden were substantially less in LDS syringes with permanently attached needles than in any of the other designs. Unfortunately, commonly available LDS syringes with permanently attached needles are 1 mL or smaller capacity and do not meet the needs of PWID who inject larger volumes of fluid. As expected, needle and syringe design (HDS vs. LDS) was the most important factor in determining dead space in a needle and syringe combination, accounting for about 77% of the variance in dead space volume. In HDS syringes with HDS needles, barrel capacity was the most important determinant of dead space, accounting for 26% of the variance in dead space volume. The contribution of barrel capacity to dead space appears to be related to the lack of standards for the height and bore of syringe nozzles. The nozzles on 10-ml and larger syringes are noticeably taller/longer than nozzles on 5-ml and smaller syringes. The bore of syringe nozzles also varies noticeably from one manufacturer to another. The tips of syringe nozzles all have the same outside diameter and the same 6° Luer taper. Standard needles are designed to these specifications, which makes standard needles and syringes interchangeable. These variations in syringe nozzle height and bore have important implications for the effectiveness of LDS detachable needles in reducing dead space. These needles have a spike that fits inside the syringe nozzle that is designed to eliminate the dead space in the nozzle of the syringe and the hub of the needle. These variations make it virtually impossible to design LDS detachable needles that fit on all syringes and achieve acceptably low dead space. Setting standards for syringe nozzle height and bore would make it possible to manufacture very LDS detachable needles that could replace standard needles. This would reduce medication waste [39–41] and dosing errors [42] due to dead space in addition to reducing HIV and HCV transmission risk among PWID. The Monoject™ LDS needles that we tested achieve low dead space when they are attached to Monoject™ syringes. However, the Monoject™ LDS detachable needles will not fit on all syringes and they are quite expensive ($0.23 apiece in lots of 1000) and available primarily through online vendors. In 2013, Exchange Supplies brought Total Dose™ LDS detachable needles to the market at a much lower price (about $0.03 apiece in lots of 1000) [18]. The dead space in Total Dose™ LDS needles attached to HDS syringes ranges from 17 μL in the new 25-mm 25-gauge needles to 54 μL in the earlier 25-mm 25-gauge needles [43]. Exchange Supplies developed these needles specifically for NSP.

Results of this study may help NSP select appropriate LDS needles and syringes to distribute. After NSP selects LDS needles and syringes, they must identify and implement strategies that assure uptake of the LDS equipment by their clients. A recent qualitative study of NSP clients and staff members in the UK concluded that LDS needles were likely acceptable to clients [44]. The study recommended offering both types of needles and implementing an intervention to promote use of LDS needles and syringes. A study that worked with NSP in two cities in Tajikistan to switch their clients from HDS needles to LDS needles provides evidence that this approach can be effective [45]. Findings from focus groups conducted during the formative phase guided the selection of low dead space needles and the development of a marketing flyer. NSP personnel in each city enrolled 100 participants and gave them low dead space needles along with a theory-based marketing flyer that emphasized the relative advantages of the needles. At a 2-month follow-up interview, 100% of participants reported trying the low dead space needles and 96% reported liking them. Both needle and syringe programs completely exhausted their supplies of low dead space needles—25,000 per city—within the first 60 days of the project, indicating wide acceptance of the needles even among clients who did not participate in the research. The findings indicate that low dead space needles are acceptable to needle and syringe program clients in these cities.

Findings from this study may also be relevant to supervised injection facilities (SIFs). These facilities have been developed to promote safer injection practices and connect people who use drugs with health and other external services [46]. Recent report by the European Monitoring Centre on Drugs and Drug Addiction (EMCDDA) suggests that there are more than 80 safe injection facilities across the globe and their number has been growing [47]. SIFs can play an important role in promoting LDS needles and syringes and facilitating acceptance by the clients.

### Limitations

We only contacted NSP in Europe and Asia, so these findings may not be generalizable to NSP in other regions that may offer needles and syringes from different manufacturers.

These data were collected in 2009 and 2010. At the time, research suggested that the dead space in HDS syringes (1 mL) with HDS needles substantially increased the probability of HIV survival and transmission risk relative to LDS syringes with permanently attached needles. Also, the differences in dead space volume among HDS syringes of different barrel capacities seemed unimportant. However, since that time, the two additional options for reducing dead space have become more widely available (Fig. 1c, d).

Finally, it was beyond the scope of this research to propose specific standards for the syringe nozzle height and bore, as this will require discussion and agreement among a broad group of stakeholders that includes industry representatives, the International Standards Organization, the WHO Safe Injection Global Network, and other multilateral international organizations. Nevertheless, we believe these findings are relevant to NSP, other harm reduction service providers, and researchers.

## Conclusions

The findings in this paper provide critical information for NSPs around the world that are in the process of selecting LDS needles and syringes to distribute. The WHO recommendation for NSP to offer LDS syringes is based on LDS 1-mL insulin syringes with permanently attached needles [7, 11]. These will not be acceptable to PWID who inject volumes of fluid greater than 1 mL. It may also be difficult to get PWID who prefer detachable needles to use syringes with permanently attached needles. This paper provides guidance regarding the potential effects of the different LDS designs. As NSPs begin implementing the WHO recommendation, this information will help them select the needle and syringe combinations that are likely to have the greatest impact on HIV and HCV transmission while still being acceptable to their clients.

## Additional file

Additional file 1: Dead space syringe project questionnaire. (PDF 668 kb)

## Abbreviations

HCV: Hepatitis C virus
HDS: High dead space
HIV: Human immunodeficiency virus
LDS: Low dead space
mL: Milliliter
mm: Millimeter
NSP: Needle and syringe program
PWID: People who inject drugs
S.D.: Standard deviation
UNAIDS: Joint United Nations Programme on HIV/AIDS
WHO: World Health Organization
μL: Microliter
