# Supplementary material for: Options for reducing HIV transmission related to the dead space in needles and syringes
Source: Harm Reduct J. 2018 Jan 15;15:3. doi: 10.1186/s12954-017-0207-5 (PMC5769352; doi:10.1186/s12954-017-0207-5)
Supplement: Additional file 1: — Dead space syringe project questionnaire. (PDF 668 kb) [file 12954_2017_207_MOESM1_ESM.pdf]

Submit by E-mail | Save | Reset Form | Print Form

Dead-Space Syringe Project Questionnaire

RTI International*
(Principal Investigator: William A. Zule, DrPH)

Please complete this questionnaire and send to Curtis Coomes at RTI International:

E-mail: ccoomes@rti.org **Fax:** 1-919-485-5555

Mail: 3040 Cornwallis Road, RTP, NC 27709, USA

Contact Information:

**Name:**			
**Organization:**			
**Type of Organization:**	☐ Syringe/Needle Exchange<br>☐ Hospital/Health Clinic<br>☐ University/Research Institution<br>☐ Pharmacy<br>☐ Other:		
**Mailing Address:**			
**City:**		**Country:**	
**Telephone:**		**Email:**	

*RTI International is a trade name of Research Triangle Institute

Syringe Information

Please answer the following questions to the best of your ability about the types of syringes and needles used by injection drug users (IDUs) in your city. If the information is not available, please answer the questions as best you can or leave the response blank.

Note: If you are ***not*** *sending us samples of your syringes, please also include a digital photograph AND a detailed description of all needles and syringes available at your location. If possible, please e-mail your pictures and survey to us at* ccoomes@rti.org. *Thank you.*

Please check the correct box for your response:

	Yes	No
1. Do any IDUs in your city or area use syringes with removable needles? **[If no, go to Question 4** (skip Questions 2 and 3)]	☐	☐

	None or almost none	Less than half	More than half	All or almost all
2. If yes…About what proportion of IDUs use syringes with removable needles? **[If none, go to Question 4** (skip Question 3)]	☐	☐	☐	☐

3. If yes…What sizes of syringes with removable needles do they use? ***Check all that apply.***

	Yes	No	Proportion distributed (for example, 10%, 50%, etc.)
1 milliliter (ml)	☐	☐	
2 ml	☐	☐	
3 ml	☐	☐	
5 ml	☐	☐	
10 ml	☐	☐	

Other (*please describe*): ____________________

4. What lengths of needles do they use? ***Check all that apply.***

	Yes	No	Proportion distributed (for example, 10%, 50%, etc.)
9.5 millimeter [mm] or 3/8 inch	☐	☐	
12.7 mm or 1/2 inch	☐	☐	
15.9 mm or 5/8 inch	☐	☐	

Other (*please describe*): ____________________

4a. What diameter needles do they use?
Check all that apply.

	Yes	No
0.51 mm (or 25 gauge or G25)	☐	☐
0.46 mm (or 26 gauge or G26)	☐	☐
0.41 mm (or 27 gauge or G27)	☐	☐
0.36 mm (or 28 gauge or G28)	☐	☐
0.33 mm (or 29 gauge or G29)	☐	☐
0.30 mm (or 30 gauge or G30)	☐	☐

	Yes	No
5. Do any IDUs in your city or area use syringes with permanently attached needles? [**If no, go to Question 8** (skip Questions 6 and 7)]	☐	☐

	None or almost none	Less than half	More than half	All or almost all
6. If yes…About what proportion of IDUs use syringes with permanently attached needles?	☐	☐	☐	☐

7. If yes…What sizes of syringes with permanently attached needles do they use?
Check all that apply.

	Yes	No	Proportion distributed (for example, 10%, 50%, etc.)
0.3 ml	☐	☐	
0.5 ml	☐	☐	
1 ml	☐	☐	
2 ml	☐	☐	

8. Syringe description: please describe the syringes available to IDUs at your location.

[*If you are sending samples to us, feel free to leave this space blank.]* Please include the following (if known) in your description: manufacturer and/or supplier, model number, syringe barrel capacity (1 ml, 3 ml, etc.), syringe barrel markings and measurements, and anything else that may help us identify which syringes are available to IDU's.

9. What factors go into deciding which particular syringes to offer IDUs in your city?

	Yes	No
Cost	☐	☐
Popularity among IDUs	☐	☐
Availability of syringes at local supplier	☐	☐

Briefly describe the factors (either listed above or others) that influence which syringes you offer in your area.

10. How many syringes of each type do you distribute annually or monthly? How many are returned?

Annually ____ or Monthly ____

Returned ____

11. How many IDUs are reached by your syringe exchange services annually or monthly?

Annually ____ or Monthly ____

12. How many IDUs are there in your city or region? ____

13. How many times per day or week do most IDUs that you encounter inject? (For example, twice a day, three times per week, etc.)

Per day ____ or Per week ____

14. Is there anything else we should know about injection behaviors in your city that may influence the number of syringes or type of syringe most IDUs in your area use in a given time period? (For example, a specific type of drug is very popular and requires unique equipment, etc.)

HIV/AIDS Information and Prevention Services

Please answer the following questions to the best of your ability about the information and prevention services you offer in your city. If the information is not available, please answer the question as best you can or leave the response blank.

15. What is the HIV prevalence among IDUs in your city or area? ☐ %

	Decreasing	Staying the same	Increasing
16. Do you think HIV prevalence among IDUs in your area is…	☐	☐	☐

Syringe access questions

	Yes	No
17. Can IDUs legally purchase syringes from pharmacies in your city?	☐	☐
18. Can IDUs legally carry syringes in your city?	☐	☐
19. Are there syringe or needle exchanges in your city?	☐	☐

Substance abuse treatment questions

20. Please check the appropriate box indicating if each type of substance abuse treatment is or is not available in your city:

	Available	Not available
Methadone maintenance	☐	☐
Buprenorphine treatment	☐	☐
Other type of opioid replacement therapy	☐	☐
Methadone detoxification	☐	☐
Outpatient drug free counseling	☐	☐
Residential treatment (less than 6 months)	☐	☐
Inpatient treatment (1 to 6 months)	☐	☐

Types of drugs injected

21 Please check the appropriate box indicating which drugs are commonly or not commonly injected in your city:	**Commonly used**	**Not commonly used**	**Percentage of users (for example, 10%, 50%, 90%)**
Opiates purchased in powder form (e.g., heroin)	☐	☐	
Opiates purchased in liquid form (e.g., kompot, shirka)	☐	☐	
Amphetamines purchased in powder form	☐	☐	
Amphetamines purchased in liquid form	☐	☐	
Methamphetamine purchased in powder form	☐	☐	
Methamphetamine purchased in liquid form	☐	☐	
Cocaine	☐	☐	

Other drugs (for example, crushed and dissolved prescription medication):

22. Where do the majority of IDUs obtain their syringes in your area?

- ☐ Pharmacies
- ☐ Other drug users
- ☐ Black market
- ☐ Syringe or needle exchanges (please note whether there are mobile units, vending machines or drop-in centers):

23. What other services do you offer besides syringe exchange to IDUs accessing your services? (For example, treatment, pamphlets, condoms, HIV testing, etc.)

24. Is there anything else about HIV or IDUs in your city that may help us with our study? (Please refer to the attached project Fact Sheet for more information about the Dead-Space Syringe Project.)

Thank you very much for your time and effort in completing this questionnaire. The results will be very important in helping to stop the spread of HIV and other blood-borne diseases through injection drug use.

Submit by E-mail | Save | Reset Form | Print Form
